# *Stenotrophomonas maltophilia *resistant to trimethoprim – sulfamethoxazole: an increasing problem

**DOI:** 10.1186/1476-0711-5-23

**Published:** 2006-09-18

**Authors:** Asma Marzouq Al-Jasser

**Affiliations:** 1 MBBS, King Saud University Fellowship of Pathology (Microbiology), Jordanian Board (Microbiology & Immunology), Certification Board in Infection Control and Epidemiology(CIC), Consultant Microbiologist, Department of Microbiology. Armed Forces Hospital, Box X-966, P.O. Box 7897. Riyadh – 11159, Saudi Arabia

## Abstract

*Stenotrophomonas maltophilia *(*S. maltophilia*) has recently emerged as an important nosocomial pathogen. Treatment of invasive infections caused by this organism is difficult as the bacterium is frequently resistant to a wide range of commonly used antimicrobials. Trimethoprim-sulfamethoxazole (TMP – SXT) is recommended as the agent of choice for the treatment of *S. maltophilia *infections. However, the development of resistance to this antibiotic represents a real challenge to laboratorians and clinicians.

This letter describes the first isolation of *S. maltophilia *resistant to TMP – SXT from two patients treated at Riyadh Armed Forces Hospital which is a major tertiary hospital in Saudi Arabia.

## Background

*S. maltophilia *is becoming increasingly recognised as an important nosocomial pathogen [1,2]. The increase is most likely due to an increase in the patient population at risk because of the advances in medical therapeutics that include: the aggressive treatment of malignancy, the increase in invasive therapeutic devices and the increased utilization of broad – spectrum antimicrobials [3]. *S. maltophilia *has emerged as a significant cause of morbidity and mortality in cancer patients [3,4]. In severely ill patients, *S. maltophilia *causes a wide range of infections such as bacteremia, pulmonary infections, urinary tract infections, wound infections, meningitis and endocarditis [2-4]. Treatment of invasive *S. maltophilia *infections is difficult because this pathogen shows high levels of intrinsic or acquired resistance to different antimicrobial agents thus drastically reducing the antibiotic options available for treatment [1,3,5]. The selection of agents for use in the management of infections due to *S. maltophilia *represents a challenge to laboratorians and clinicians because of the problems associated with *in vitro *susceptibility testing, the inherent resistance of the bacterium to many antimicrobial agents and the limited clinical trials to determine the optimal therapy [6-8]. In *vitro *susceptibility testing of *S. maltophilia *must be interpreted with caution. For most antibiotics, inconsistent results were obtained from different susceptibility testing methods [9]. Disc diffusion methods are deemed inaccurate and of poor reproducibility as the agar composition and the duration of incubation may influence the results. Quinolone agents, particularly ciprofloxacin, appear most problematic in this respect [3,9]. The National Committee of Clinical Laboratory Standards (NCCLs) currently recommends testing for minimal inhibitory concentrations (MICs) by the use of the agar or the broth dilution method [8].

TMP – SXT has been recommended for use in the treatment of *S. maltophilia *infections based on the *in vitro *susceptibility data which confirm its high activity and the favourable outcomes observed in patients treated with this agent [3,6]. Although the role of the combination antimicrobial therapy in treating infections due to strains that are susceptible to TMP – SXT is uncertain but the addition of one or more agents to which the isolate is susceptible in *vitro *is a reasonable consideration if the patient is critically ill or has an underlying haematological malignancy [4,6,7]. Several reports have shown that the prevalence of strains that are resistant to TMP – SXT is increasing [3-5,7,10,11]. The rate of resistance to TMP – SXT ranges from 2% in Canada and Latin America to 10% in Europe [10]. Reported here are two cases of *S. maltophilia *infection which were found to be resistant to TMP – SXT.

In the first case, *S. maltophilia *was isolated on 16/7/2005 from a 48 years old Saudi female. She received a course of cytotoxic chemotherapy to control the blast cell transformation of her chronic myeloid leukemia. Thereafter, she was given a three week course of intravenous antibiotics (meropenem 1 gram every 8 hours, gentamicin 2 mg/kg twice daily and vancomycin 1 gram every 12 hours) for the treatment of febrile neutropenic episodes. Then she was admitted to general intensive care unit (GICU) with septic shock. Meanwhile, a bone marrow aspirate showed no response to the course of chemotherapy given. After her death, a blood culture set taken from the central venous catheter grew *S. maltophilia*. No organism was isolated from the other set of blood taken simultaneously via a percutaneous venipuncture. The Hickman catheter tip culture showed no growth.

The second organism was isolated on 10/10/2006 from a 65 years old Saudi male who had carcinoma of the urinary bladder with end stage renal disease. He was admitted to the urology ward due to obstructive uropathy. Bilateral percutaneous nephrostomy tubes and a uretheral catheter were introduced to relieve the obstruction. Prior to admission, he received oral norfloxacin 400 mg twice daily for 3 days. After admission, norfloxacin was replaced by intravenous ceftriaxone 2 grams once daily for one week. A urine specimen collected from the left percutaneous nephrostomy tube grew *S. maltophilia*. He was clinically stable with no local signs of infection. The cultures of urine specimens from both the right percutaneous nephrostomy tube and the uretheral catheter were negative. Both nephrostomy tubes were changed later and the repeated urine cultures showed no growth. Both organisms were identified with 99% confidence by the Analytical Profile Index (API) 20 NE (Biomeriux, France). The sensitivity testing was done by the automated MicroScan system. Interpretation of the MICs was based on the guidelines of the NCCLs [8]. Both isolates were resistant to TMP – SXT (MIC > 8/152 ug/ml by MicroScan system and MIC > 32 ug/ml by Etest strip). The two isolates were also resistant to gentamicin (MIC > 8 uglml), both meropenem and imipenem (MIC > 16 ug/ml) and ciprofloxacin (MIC > 4 ug/ml). They were sensitive to ceftazidime (MIC < 2 ug/ml) and ticarcillin – clavulanate (MIC = 16/2 ug/ml). The sensitivities to amikacin, chloramphenicol, tetracycline, levofloxacin, aztreonam and piperacillin – tazobactam were variable between the two isolates.

Several studies have recommended the consideration of the use of TMP – SXT as the initial agent of choice for the treatment of serious *S. maltophilia *infections [6,7]. The addition of another antimicrobial agent to the initial regimen should be considered if there is a significant incidence of resistance to TMP – SXT among the isolates in a particular facility [5-7,9]. Other useful agents which have demonstrated in *vitro *activity against *S. maltophilia *include: some quinolones (levofloxacin, gatifloxacin, moxifloxacin and clinifloxacin), tygecyclin, polymyxins (colistin and polymyxin-B) and rifampicin [11-13]. Variations have been observed with different synergestic studies (broth and agar dilutions, checkerboard and half-checkerboard techniques as well as time-kill curves). Combinations identified to be synergestic by the checkerboard method were not found to be so by killing curve criteria [3,11]. Studies have shown that non-synergestic in *vitro *combinations have been associated with successful therapeutic outcomes [3,5]. Also, it is important to note that synergy may not occur at clinically achievable concentrations [15,16]. Therefore, it is difficult to draw firm conclusions from different in *vitro *synergistic studies not only because of different methodology used and wide combinations tested but also because of strain to strain variation since synergy may be strain dependent [3,14-16]. Randomized trials on potentially efficacious agents singly and in combinations are warranted. The antibiogram of both of our isolates suggests a possible role of other antimicrobials eg. ceftazidime, ticarcillin – clavulanate.

The isolation of *S. maltophilia *which is resistant to TMP – SXT at our institution is alarming. The proposed strategies to prevent *S. maltophilia *infection should be encouraged and they include: the avoidance of inappropriate use of antibiotics, the avoidance of prolonged implementation of foreign devices, the reinforcement of hand hygiene practices and the application of appropriate infection control practices. The microbiology laboratory also plays a vital role in controlling *S. maltophilia *infections by continuous monitoring of the prevalence, the provision of local antibiogram data and the performance of synergistic studies which may help to guide therapy selection.
